# Scrt1, a transcriptional regulator of β-cell proliferation identified by differential chromatin accessibility during islet maturation

**DOI:** 10.1038/s41598-021-88003-2

**Published:** 2021-04-22

**Authors:** Jonathan Sobel, Claudiane Guay, Ofer Elhanani, Adriana Rodriguez-Trejo, Lisa Stoll, Véronique Menoud, Cécile Jacovetti, Michael D. Walker, Romano Regazzi

**Affiliations:** 1 Department of Fundamental Neurosciences, University of Lausanne, Rue du Bugnon 9, 1005 Lausanne, Switzerland; 2 Department of Biomolecular Sciences, Weizmann Institute of Science, 7610001 Rehovot, Israel; 3 Department of Biomedical Sciences, University of Lausanne, Rue du Bugnon 7, 1005 Lausanne, Switzerland; 4 Present Address: Department of Medicine, Weill Cornell Medicine, 413 East 69th Street, New York, NY 10021 USA

**Keywords:** Computational biology and bioinformatics, Molecular biology, Physiology, Endocrinology

## Abstract

Glucose-induced insulin secretion, a hallmark of mature β-cells, is achieved after birth and is preceded by a phase of intense proliferation. These events occurring in the neonatal period are decisive for establishing an appropriate functional β-cell mass that provides the required insulin throughout life. However, key regulators of gene expression involved in functional maturation of β-cells remain to be elucidated. Here, we addressed this issue by mapping open chromatin regions in newborn versus adult rat islets using the ATAC-seq assay. We obtained a genome-wide picture of chromatin accessible sites (~ 100,000) among which 20% were differentially accessible during maturation. An enrichment analysis of transcription factor binding sites identified a group of transcription factors that could explain these changes. Among them, Scrt1 was found to act as a transcriptional repressor and to control β-cell proliferation. Interestingly, *Scrt1* expression was controlled by the transcriptional repressor RE-1 silencing transcription factor (REST) and was increased in an in vitro reprogramming system of pancreatic exocrine cells to β-like cells. Overall, this study led to the identification of several known and unforeseen key transcriptional events occurring during β-cell maturation. These findings will help defining new strategies to induce the functional maturation of surrogate insulin-producing cells.

## Introduction

Pancreatic β-cells are highly specialized cells displaying the unique functional feature to release insulin in response to glucose and other stimuli. Inability of β-cells to secrete the appropriate amount of insulin to cover the organism’s needs leads to diabetes mellitus. β-cells acquire the ability to secrete insulin through a poorly understood postnatal maturation process involving transcriptional reprogramming of gene and non-coding RNA expression that becomes complete only after weaning^1,2^. In rats, this process occurs at around 21 days after birth^1,3,4^. Cells that produce and secrete insulin can be generated in vitro using various methods^5,6^ and are similar to β-cells in many aspects. However, these cells show lower glucose-stimulated insulin secretion (GSIS) and transcriptome differences compared with normal β-cells^7^. Consequently, there is a need to understand better the regulation of the maturation process to enable the engineering of fully functional surrogate insulin-producing cells. Likewise, the transcriptional control of pancreas development and islet differentiation remains a subject of intensive investigations^8–11^. In the past decade, the emergence of various next-generation sequencing technologies and experimental procedures have allowed querying of epigenome and gene expression profiles with unprecedented precision at a genome-wide or transcriptome-wide scale. Indeed, Ackermann et al. identified human *α* and β-cell specific mRNAs and regulatory elements using RNA-seq and ATAC-seq, respectively^12^. The ATAC-seq method probes DNA accessibility with a hyperactive Tn5 transposase, which inserts sequencing adapters into accessible chromatin regions. Sequencing reads can then be used to infer regions of increased accessibility, as well as to map regions of transcription factor binding and nucleosome position^13^. This allows the identification of transcription factors (TFs) that will bind to specific binding sites (TFBS) located in an open chromatin region, which can be close or distant to the transcription start site (proximal/distal regulation). Moreover, sets of TFs can cooperate on cis-regulatory modules (CRM), which are DNA stretches of about 100–1000 bp, to produce specific regulatory events^14^.

Taking advantage of this approach, we aimed at determining how neonatal islet maturation in rat is controlled at the transcriptional level, and to identify the key transcription factors involved. Open chromatin regions in the islets of 10-day-old (P10) and 3-month-old (adult) rats were analysed by ATAC-seq. About 100,000 putative regulatory regions were identified using this method, 20% of which displayed significant accessibility changes upon maturation. Using two different computational approaches to investigate potential TFBS motifs in the regions with accessibility changes, we identified putative regulatory elements in the promoter or in distal CRM of genes encoding differentially expressed mRNAs that may be implicated in islet maturation. As a result, a global picture of the transcriptional events taking place during pancreatic islet maturation was obtained. The involvement in post-natal β-cell maturation of a novel transcriptional regulator, Scrt1, identified in our computational analysis was confirmed experimentally. *Scrt1* expression was found to be controlled by the transcriptional repressor RE-1 silencing transcription factor (REST) and to increase during the course of reprogramming of pancreatic exocrine cells into β-like cells^15^.

## Results

**Figure 1:**
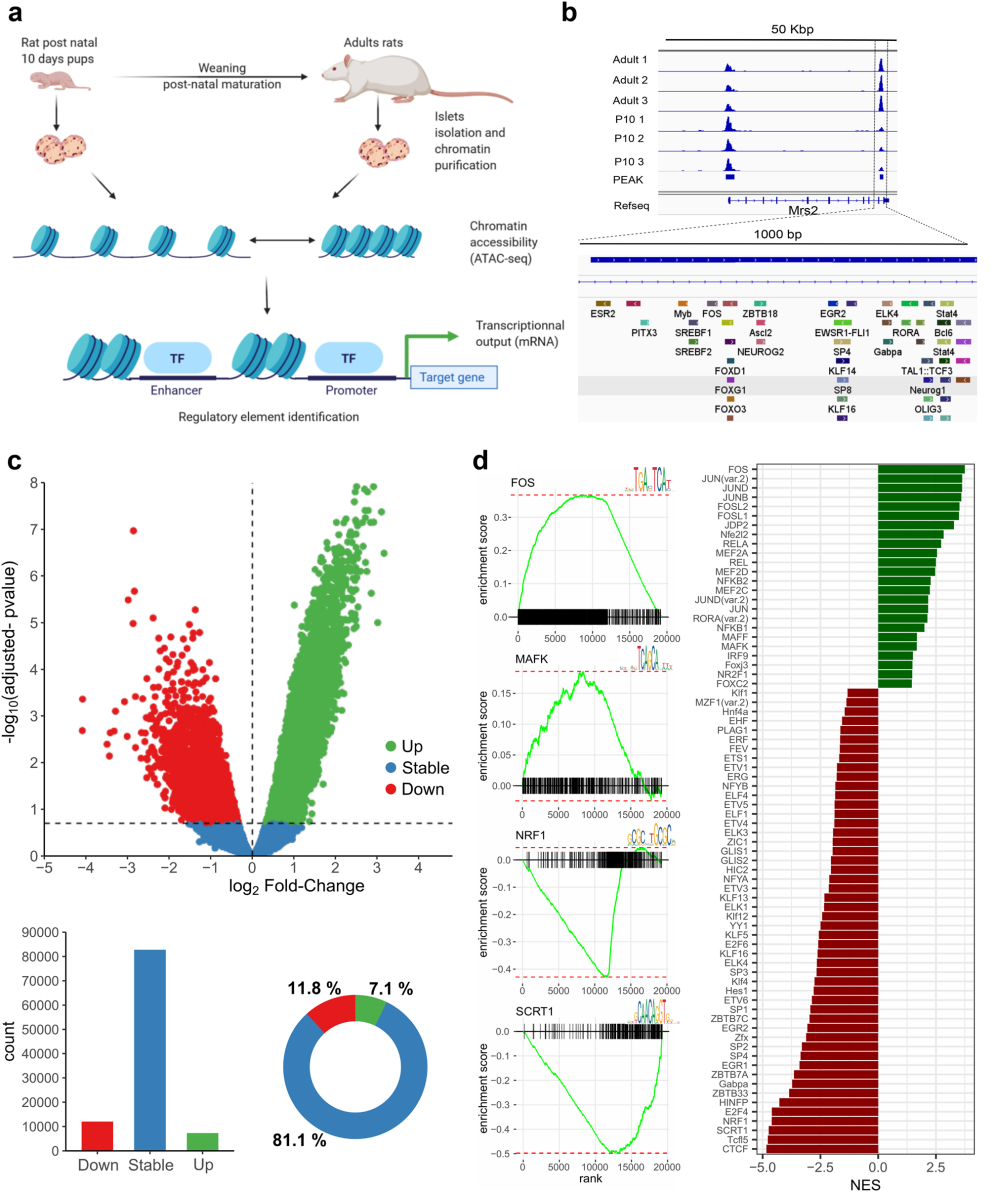
ATAC-seq successfully identified accessible sites (ACS) and transcription factor binding sites (TFBS) motif associated with pancreatic islet maturation. (**a**) Summary of the experimental design. Nuclei were extracted from the islets of 3 adult rats and 3 litters of rat pups at postnatal day 10 (P10) to perform the Tn5 reaction as described in^51^ and prepare the library for sequencing. The computational pipeline involved a quality control of the sequencing data followed by read alignment to the rat reference genome (Rn5 assembly). ACS were identified using the peak calling tool MACS2^17^ and quantified for each sample separately. The ACS sequences were scanned using FIMO^20^ and analyzed to identify TFBS motifs that are implicated in the islet maturation process and in related pathways (See methods). (**b**) Example of identified ACS. The ACS nearby *Mrs2* transcription end site is significantly higher in adult rats. This ACS contains several TFBS motifs. (**c**) Differential analysis of Trn5 integrations in accessible sites. On the top, the volcano plot representation of the log_2_ fold-change of Trn5 integration between P10 and adult rat islets in the x-axis, and the FDR adjusted p-value in the y-axis. ACS more accessible in adults are represented in green (Up), those more accessible in P10 in red (Down), and those remaining stable in blue (p-value < 0.05, FDR < 0.2, n = 3). Below, the barplot of the number of ACS changing along postnatal maturation and the donut plot of the percentage of Up, Down and stable ACS. (**d**) Motif enrichment analysis in ACS identified putative regulators of postnatal pancreatic islet maturation. The left panels represent four enriched TFBS motifs. The right panel represents the FGSEA normalized enrichment score for top enriched motifs (FGSEA adjusted p-value < 0.05). See also Supplementary Figure S1 and Supplementary Table 1,2,3.

To study the transcriptional regulation of pancreatic islet maturation, we performed high throughput sequencing of transposase-accessible chromatin of newborn and adult rat islets (Fig. 1a). Post-natal β-cell maturation in rat has been shown to occur on a slightly different time frame compared to mice^3,4,16^. Consistent with the data in the literature we previously observed that P10 rat β-cells have a high proliferative capacity compared to adult mature β-cells but are unable to secrete insulin in response to glucose^1^. Therefore, we elected to compare chromatin accessibility in 10 days-old rat islets (P10) and in adult rat islets. Bioinformatic methods for reads alignment, quality control, peak detection, differential accessibility analysis and motif finding were applied. For example, the Magnesium Transporter 2 (*Mrs2*) locus depicts two prominent ATAC-seq signal peaks, and the 3′ end peak shows an important increase of accessibility after the maturation process (Fig. 1b). Quality control of the samples (Table 1) showed an average of 370 million reads sequenced with 94.3% of reads with a MAPQ score above 30. The analysis of fragment size distribution showed the expected profile of ATAC-seq (Supplementary Fig. S1a) with usual oscillations due to the presence of nucleosomes. In addition, adult and P10 samples were well clustered, when evaluated by the correlation between samples (Supplementary Fig. S1b) or using the first PCA component (Supplementary Fig. S1c). To detect accessible sites (ACS), we performed a peak calling using MACS2^17^ (Methods), leading to the detection of ~ 102,000 ACS (Supporting Information 1). These sites were quantified in each sample separately and annotated with the closest transcription start site (TSS). Moreover, a differential accessibility analysis was performed using EdgeR^18^ (Fig. 1c, top), and the values of the analysis reported in the source data (1). ACS were divided in three groups: Stable, significantly more accessible in P10 (Down) and significantly more accessible in adults (Up), with p-value < 0.05 and FDR < 0.2. About 20% of the ACS showed differential accessibility upon maturation with 11.8% of down ACS and 7.1% of up ACS (Fig. 1c, bottom).

**Table 1:** ATAC-seq libraries quality control.

Sample	Total reads in library	Percentage with MAPQ ≥ 30
A2_4	390,125,344	96.6
A2_5	363,039,104	94.8
A2_6	319,607,584	95.7
P2_1	366,722,400	93.7
P2_2	320,852,352	94.1
P2_3	447,404,512	91.2

### Promoter and distal ACS depicted distinct accessibility patterns along islet maturation

To assess the impact of the ACS location in respect to the closest gene, we used ChIPseeker^19^ to annotate our ACS and 100,000 randomly selected sites (Supplementary Fig. S1d). ACS were enriched for exonic and intronic sequences and transcript start sites as opposed to random sites (Supplementary Fig. S1e). Interestingly, ACS more accessible in adults (up), were enriched in distal sites and intron as compared to ACS less accessible in adults (down), which were enriched in promoters. We observed that 2/3 of the ACSs were distal sites and about 10% were located in the TSS or proximal class.

### Identification of transcriptional regulators and chromatin remodelers of pancreatic islet maturation

Accessibility analysis enables the detection of TFs or TFBS affecting the chromatin state and the transcription of nearby genes. In order to decipher the combinatorial code of transcription factor binding sites that allows islet maturation, we scanned the sequence of each ACS using FIMO^20^ together with the position weight matrices of Jaspar 2016^21^. With these sequence scans we could perform a motif set enrichment analysis for the accessibility in P10 or in adult islet cells using the FGSEA algorithm^22^ (Supplementary Table 2, Fig. 1d). Thus, using the set of significantly changing ACS and their respective accessibility log_2_ Fold Change, we were able to identify TFBS motifs that were either enriched in P10 or in adult rat islets. Several TFBS, previously implicated in islet maturation, were significantly enriched, such as MAF, FOX, FOS/JUN, NRF, and E2F^23–28^. Moreover, several motifs recognized by transcriptional repressors and insulators such as SCRT1 or CTCF were also enriched in P10 islets. To confirm these results and detect additional motifs playing a role in islet maturation, we applied a penalized linear model GLMnet^29^ to all ACS with the matrix of motif match as predictors and the log_2_ fold-change as output vector. With this method, an activity (β) for each motif was computed (Supplementary Table 3). This allowed to confirm most of the hits discovered using the FGSEA and to detect additional TFBS motifs such as RFX, SREB, NKX6, REL, MEIS, and TEAD3.

**Table 2:** qPCR primer sequences (rat).

	Forward	Reverse
Cnb1	5′-TGCTTCAGGAGGGACTGACT-3’	5′-CCACCTCCCTCACACAAACT-3’
Glucagon	5′-GAAGTTACCGCCCTGAGATT-3’	5′-CGCATTTATGACAAAGGGTTC-3’
Hprt	5′-AGTCCCAGCGTCGTGATTAG-3’	5′-AATCCAGCAGGTCAGCAAAG-3’
Insulin2	5′-TGGGGAGCGTGGATTCTTCT-3’	5′-CAGAGGGGTGGACAGGGTAG-3’
MafB	5′-TATTCCAAGGAGTCGCCAAG-3’	5′-CTGAGAGCCAGTGTTCACCA-3’
NeuroD1	5′GGATGATCAAAAGCCCAAGA-3’	5-GCAGGGTACCACCTTTCTCA-3’
Nfatc1	5′-TTGGATTCTGACGAGCTGTG-3’	5′-GTGCAGCTGGATCAAGAACA-3’
Nfatc2	5′-CATTCCCATCTGCAGCATCC-3’	5′-CCGTCCCGATGAAGATCTGA-3’
Notch1	5′-CTATGTTGTGGACCATGGCG-3’	5′-CGGCTTGCTGACATGACTTT-3’
Pax6	5′-AGGAACCAGAGAAGACAGGC-3’	5′-GTACGAGGAGGTCTGACTGG-3’
Scrt1	5′-ACATTCTCTTCGGCAGACCT-3’	5′-GGATGGCCCTTTGAGCAATG-3’
Syt4	5′-TACCAGCCATGGATGAACAATC-3’	5′-CAAAACTCAGGACGGTGAAGTG-3’

**Table 3:** qPCR Primer Sequences (mouse).

Gene	F-primer	R-primer
Mouse Ins2	TGGCTTCTTCTACACACCCATG	AGCTCCAGTTGTGCCACTTGT
Mouse Pdx1	AGCTCACGCGTGGAAAG	CAGTACGGGTCCTCTTGTTT
Mouse Rest	AAGACTCATCTAACGCGACAC	GGGTCACTTCATGCTGATTAGA
Mouse Scrt1	GGAGTGCGACTGCAAGATAA	ACAGCCGCAGCATACATAG
Mouse Snap25	CCATCAGTGGTGGCTTCAT	TCAATCTCATTGCCCATGTCT
Mouse Tbp	ATTCCGCCTTCCCAGTATT	TCTAACAATTTACAAGCTGCGT

### ACS significantly affected were located near genes involved in islet maturation

Next, we investigated if ACS changing in P10 versus adult rat islets control the expression of genes displaying significant differences upon maturation^30^. Of the 19,311 ACS differentially accessible, 11,372 (~ 60%) were located in the vicinity of differentially expressed genes. These ACS were annotated as enhancers or repressors, depending whether their accessibility was respectively correlated or anti-correlated with the expression changes in the nearby gene (Supporting Information 1). As previously described^30^, several KEGG pathways were enriched by analyzing significantly changing genes nearby differentially accessible ACS. For instance, insulin secretion, circadian rhythm, and calcium signalling were enriched in adult, while carbon metabolism, PI3-Akt, and proliferation related annotations (cancer) were enriched in P10 (Supporting Information 1). Enhancer and repressor ACS contribute to the output gene expression. Thus, a gene may be regulated by several ACS, some bound by enhancer proteins, and others targeted by repressors.

### ACS display enhancer activity

**Figure 2:**
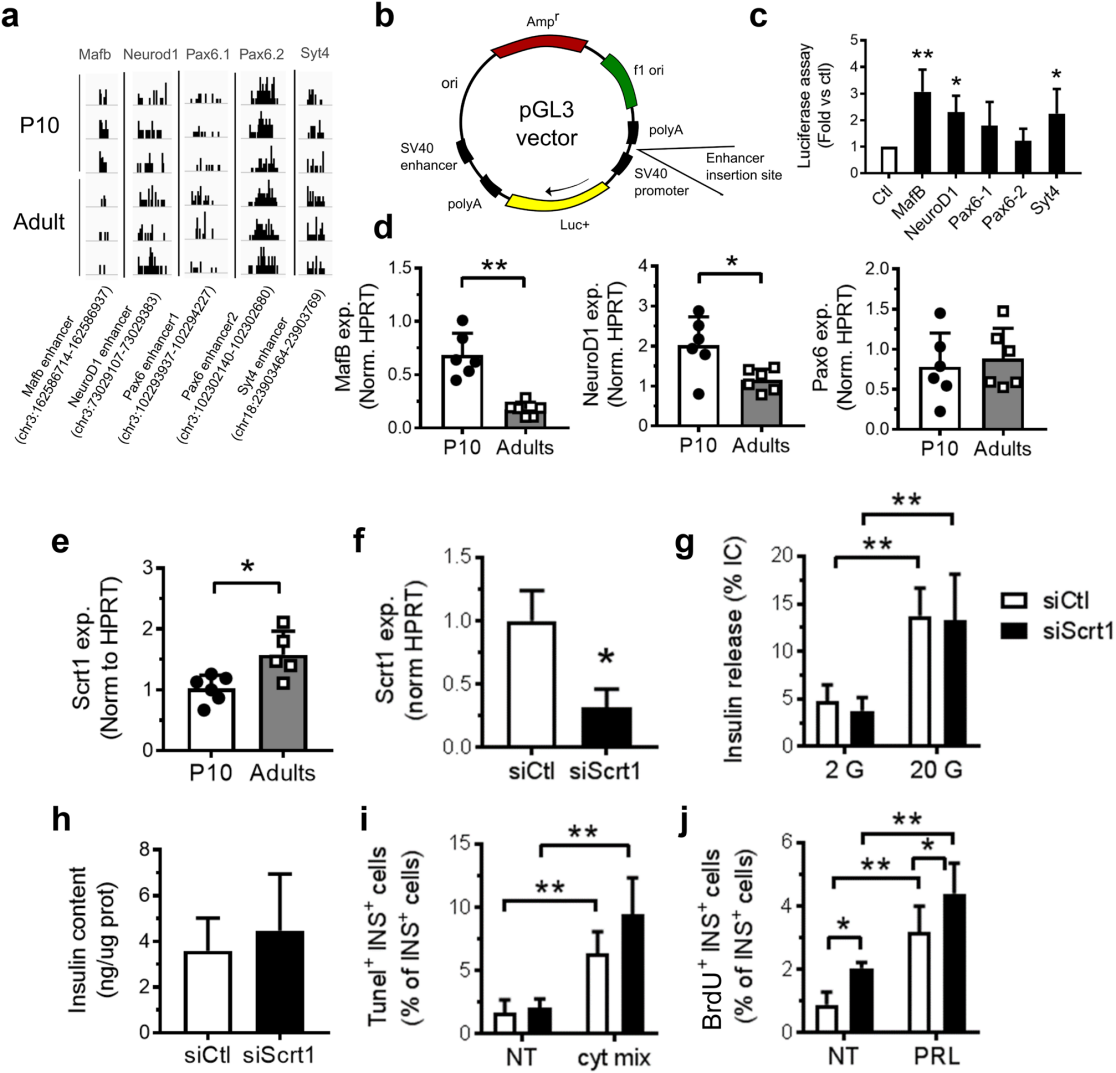
Enhancer activity assessment and Scrt1 function. (**a**) Trn5 integrations on *Mafb, Syt4, Neurod1* and two *Pax6* accessible sites. (**b**) Scheme of pGL3 vector used for the luciferase assay. (**c**) Luciferase activity was measured in INS 832/13 cells transfected with an empty pGL3 vector (Ctrl) or a pGL3 vector containing an enhancer region for the indicated gene (*Mafb, NeuroD1*, *Pax6* and *Syt4*). Results are expressed as fold change versus control. (**d**) Gene expression in P10 and adult rat islets were measured by qPCR and normalized to the housekeeping gene *Hprt1*. *Syt4* gene expression is available in Fig. 3. * p < 0.05, ** p < 0.01 by Student’s t-test or by one-way Anova, Dunnett’s post-hoc test. (**e**–**f**) *Scrt1* expression was measured by qPCR and normalized to *Hprt1* housekeeping gene levels. (**f**–**j**) Dispersed adult rat islet cells were transfected with a control siRNA (siCtl) or siRNAs directed against Scrt1 (siScrt1). Experiments were performed 48 h post-transfection. Insulin release in response to 2 or 20 mM glucose (**g,h**) insulin content were determined by ELISA n = 3. (**i**) Apoptosis of insulin-positive cells was assessed using Tunel assay in basal (NT) condition or in response to a mix (cyt mix) of pro-inflammatory cytokines (IL-1β, TNF-α and IFN-γ) n = 5. (**j**) The fraction of proliferative insulin-positive cells was determined by BrdU incorporation in basal (NT) or stimulated (prolactin, PRL) conditions, n = 6. * p < 0.05, ** p < 0.01 by Student’s t-test or by one-way Anova, Tukey’s post-hoc test. See also Source data (4) and supplementary Fig. S3.

Next, we experimentally tested if the identified ACS located near genes important for proper pancreatic β-cell function^31^ display enhancer activity. For this purpose, 5 ACS sequences (Supplementary Table 4, Fig. 2a–c) near *Syt4, Pax6* (two ACS), *Mafb* and *NeuroD1* were cloned in a luciferase reporter construct driven by a minimal promoter. Interestingly, the inclusion of the ACS close to *Syt4, MafB*, and *NeuroD1* resulted in an increase in luciferase activity, compared to the empty pGL3 vector. In addition, mRNA expression of *NeuroD1* and *MafB*, tested by qPCR (Fig. 2d), confirmed the higher expression of these genes in 10-day-old pups. MAFB was previously described as a regulator of the pancreatic β-cell function^32^ and its expression to be decreased during β-cell maturation in mice^23^ (reported in Supplementary Fig. S2b). Here we detected a cis-regulatory elements containing various TFBS motifs including Meis1,2 or Glis2 located more than 10Kbp away from the *Mafb* gene that was less accessible in adult rats (Supplementary Fig. S2a). Thus, we concluded that the identified ACS are likely to be involved in transcriptional regulation of nearby genes.

### Scrt1 represses β-cell proliferation

We next focused on Scrt1, a transcriptional repressor involved in neuroendocrine development^33–35^ but whose functions in β-cells remain to be determined. Binding sites for this transcriptional repressor were highly enriched in the chromatin regions that close upon β-cell maturation. Thus, our hypothesis was that Scrt1 binding in those sites would close the chromatin. In agreement with the lower chromatin accessibility for Scrt1 binding sites, *Scrt1* expression is increased in adult rat islets compared to immature islets (Fig. 2e, Supplementary Fig. S2d). Interestingly looking into the data-set published by Qiu et al*. *^23^*, Scrt1* expression was also found to be augmented during maturation of mouse β-cells (Supplementary Fig. S2c). Transfection of adult rat islet cells with a set of siRNAs targeting Scrt1 led to a decrease in the expression of the repressor of about 70%. (Fig. 2f). Scrt1 knockdown neither affected glucose-stimulated insulin secretion (Fig. 2g) nor insulin content (Fig. 2h). Apoptosis measured by Tunel assay in both control condition or in response to pro-inflammatory cytokines was also not affected (Fig. 2i, supplementary Fig. S3a). Interestingly, knockdown of *Scrt1* in adult β-cells resulted in a rise in proliferation, suggesting an involvement of this transcriptional repressor in postnatal β-cell mass expansion (Fig. 2j, supplementary Fig. S3b).

### Identification of Scrt1 targets involved in maturation

**Figure 3:**
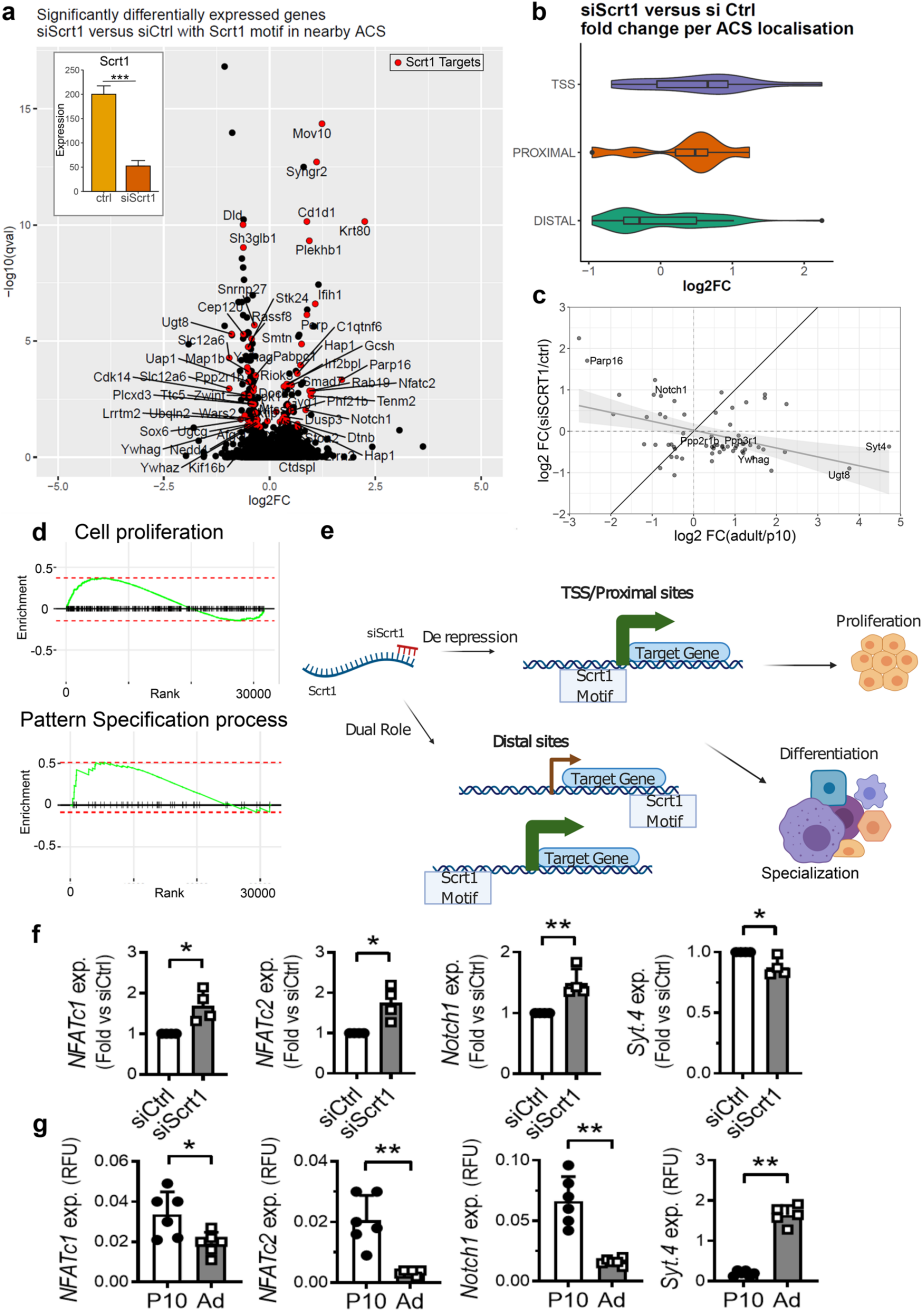
Changes induced by silencing *Scrt1* were anti-correlated with the maturation signature of islet cells. FAC-sorted adult rat β-cells were transfected with a control siRNA (siCtl) or siRNAs directed against Scrt1 (siScrt1). RNA extraction and library preparation for RNA-seq were performed 48 h post-transfection. (**a**) Volcano plot of gene expression changes induced by *Scrt1* knockdown. SCRT1 putative target genes (defined based on the presence of SCRT1 motif in ACS in the proximity of the gene) significantly altered are indicated with red dots and labels. In the top left, normalized counts from the RNA-seq data of *Scrt1* expression is represented with a barplot. (**b**) log_2_ fold changes distribution of significantly altered genes by siScrt1 containing a SCRT1 motif in an ACS either at the TSS (± 1Kbp), proximal (1-10Kbp) or distal (more than 10Kbp) from the gene. (**c**) Scatter plot of log_2_ fold changes of differentially expressed genes from adult/P10 measured by micro-array (n = 3) versus log_2_ fold changes of siScrt1/control measured by RNA-seq (n = 5). Several genes of interest are represented with their label. (**d**) Enrichment plot for two significant annotations using the FGSEA algorithm. (**e**) Scheme describing the siScrt1 hypothesis. Briefly, the siRNA for Scrt1 will reduce the mRNA level of the *Scrt1* gene and consequently the level of the protein. Thus, lower binding of SCRT1 will occur at target sites that will affect proliferation and specialization of β-cells. qPCR confirmation of gene expression in (**f**) FAC-sorted β-cells transfected with siCtrl or siScrt1 or in (**g**) P10 versus adult rat islets. Student T test, *p < 0.05, **p < 0.01. See also Supplementary Fig. S5 and Supplementary Tables 5, 6, 7, 8.

As Scrt1 appears to regulate the proliferative capacity of β-cells, we next aimed at finding its targets. For this purpose, adult rat islet cells were FACS-sorted to separate *α*-cells and β-cells. We observed that *α*-cells and β-cells express *Scrt1* at similar levels (Supplementary Fig. S4a). Subsequently, an RNA-seq on β-cells exposed to a control siRNA or to siScrt1 was performed. Differential expression analysis between siScrt1 and control samples revealed that 168 genes were significantly impacted by silencing *Scrt1* with a FDR adjusted p-value below 0.05 (Fig. 3a). Of these 168 genes, 111 were down-regulated and 57 were up-regulated. The significantly changing genes were enriched with ACS containing Scrt1 motif in their surroundings (hypergeometric test p-value < 10^–6^). We then looked at the log_2_ Fold-change distribution of ACS located at the TSS, proximal or distal (Fig. 3b). We observed that genes having a Scrt1 motif containing ACS at the TSS or proximal were increased upon siScrt1 while a substantial part of the distal Scrt1 ACS were decreased. As expected, among the potential targets of Scrt1 we found genes related to proliferation such as *Notch1, Parp16, Ppp3r1, Ppp2r1b* and *Ywhag* (Fig. 3c). Moreover, some genes related to glucose signalling and GSIS such as *Syt4*, or to sphingolipid metabolism as *Ugt8* were down-regulated when knocking down *Scrt1*. Then, we compared the set of genes affected by *Scrt1* silencing and the ones differentially expressed upon maturation (in postnatal 10-day-old (P10) versus adult rat islets). A common set of 62 genes was found to change in both data sets with an FDR adjusted p-value below 0.05 (Fig. 3c, Supplementary Table 7). In addition, a gene ontology (GO term) enrichment analysis for biological processes revealed that autophagy and oxygen sensing are over represented in these 62 genes (Supplementary Table 8). Interestingly, a significant anti-correlation (Pearson’s correlation test, p-value = 0*.*013) was observed between the fold-changes from the comparison between siScrt1 versus siCtl in adult rat β-cells and P10 versus adult islets. The opposite change in the expression of *Nfatc1*, *Nfatc2*, *Notch1* and *Syt4* in response to *Scrt1* downregulation versus islet maturation was also confirmed (Fig. 3f,g, Supplementary Fig. S5).

Next, we looked at the enriched gene ontologies using the FGSEA algorithm on the RNA-seq of siScrt1 and siCtrl. Cell proliferation and pattern specification process were among the highest hits (Supplementary Table 5, Fig. 3d).

Taken together our results suggest that Scrt1 act as a repressor that regulate proliferation through de-repression of genes at their TSS or in cis-regulatory elements, while specialization seems to occur through distinct mechanisms involving distal ACS (Fig. 3e).

### Scrt1 is a target of REST and is induced in exocrine to β-cell reprogramming

**Figure 4:**
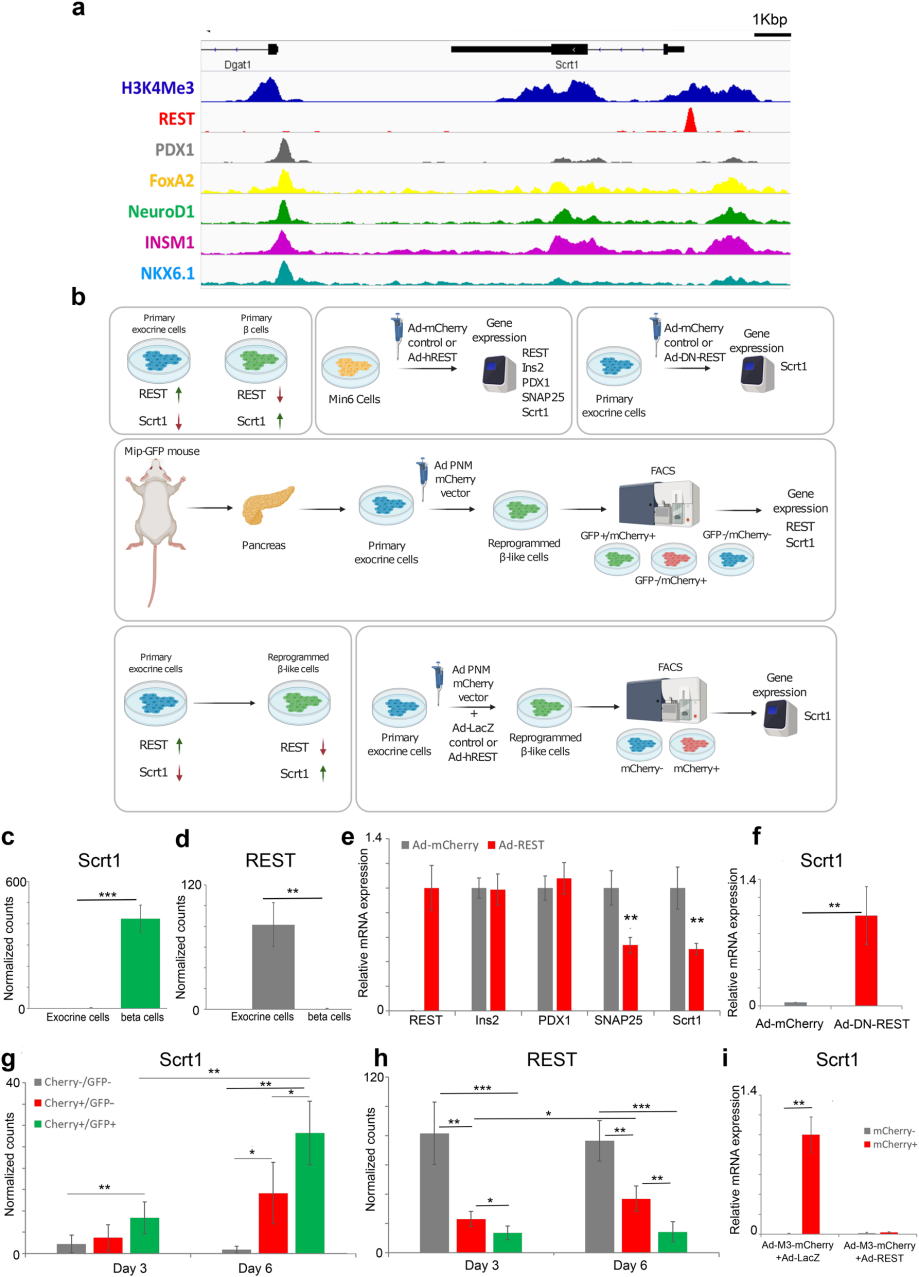
Scrt1 is negatively regulated by REST in β cells, exocrine cells and reprogrammed β-like cells. (**a**) The mouse *Scrt1* locus showing hallmark β-cell transcription factor binding sites and H3K4Me3 in β-cells and REST binding in mES cells. (**b**) Scheme describing the experiments done in (**c**–**i).** Normalized counts from RNA-seq data showing *Scrt1* (**c**) and *Rest* (**d**) expression in cultured mouse primary exocrine cells and in isolated primary β-cells. Primary exocrine cells are the mCherry-/GFP- as described in (**g**,**h)**. Primary β-cells are the GFP + FAC-sorted fraction of islets isolated from MIP-GFP mice. Results are mean normalized counts ± SD, and ***p < 0.001, n = 4. (**e**) Min6 cells were infected with Ad-mCherry control virus or Ad-hREST and 3 days post infection gene expression was measured by qPCR and normalized by housekeeping gene (*Tbp*). Results are presented as relative expression versus Ad-mCherry control and are mean ± SD; ** p < 0.001 on t-test, n = 4. (**f**) Primary exocrine cells were infected with Ad-mCherry control or Ad-DN-REST viruses and 5 days post-infection *Scrt1* expression was analyzed by qPCR and normalized by *Tbp*. Results are presented as relative expression levels versus Ad-DN-REST group and are mean ± SD; n = 4. Normalized counts from RNA-seq data showing *Scrt1* (**g**) and *Rest* (**h**) expression in reprogrammed β-like cells from primary exocrine cells. Primary exocrine cells isolated from MIP-GFP mice were infected with Ad-M3-mCherry virus. Day 3 and 6 Cherry/GFP fractions were FAC-sorted as indicated. Cherry + represents the M3-infected fraction and the GFP + fraction enriched for reprogrammed β-like cells. Results are mean normalized counts ± SD, *p < 0.05, n = 4. (**i**) FAC-sorted day 5 reprogrammed exocrine cells expressing M3-mCherry and LacZ or REST. mCherry fractions were FAC-sorted and *Scrt1* expression was analyzed by qPCR and normalized by *Tbp*. Results are presented as relative expression levels versus mCherry + fraction of Ad-mCherry + Ad-LacZ control virus group, and are mean ± SD; *** p < 0.001, n = 4. See also Supplementary Fig. S6 and Supplementary Table 9.

To gain insights in to the mechanisms underlying *Scrt1* regulation during β-cell maturation, we analysed previously published ChIP-seq data for hallmark β-cell transcription factors in mouse islets^36–38^. Interestingly, the *Scrt1* locus is bound by PDX1, FoxA2, NeuroD1, INSM1 and NKX6-1 (Fig. 4a). Moreover, this locus is marked by H3K4Me3 in islets^39^, suggesting that the *Scrt1* gene is active in the endocrine pancreas. Since REST (RE-1 silencing transcription factor) was previously reported to be an important repressor of β-cell genes that is silenced in adult islets^15,40,41^, we also reanalysed the binding of this transcriptional repressor^42^ to the *Scrt1* locus. Interestingly, REST binds the *Scrt1* locus in non-islet mouse and human cells (ES and Panc1 cells) (Fig. 4a, Supplementary Fig. S6), suggestive of a REST-mediated repression of the *Scrt1* gene. Given the identification of a REST binding site upstream of *Scrt1* gene (Fig. 4a), and a specific expression of *Scrt1* in endocrine and not in exocrine cells (Fig. 4c), as well as the recently published involvement of REST in acinar to β-cell reprogramming^84^, we sought to test the regulatory role of REST on Scrt1 expression, in this biologically relevant and well controlled reprogramming system.

To address the potential repression of *Scrt1* by REST in pancreatic cells, a series of experiments were performed on mouse exocrine, β-, and reprogrammed β-like cells, as described in Fig. 4b. First, analysis of previously published transcriptomic data^15^, shows that *Scrt1* (Fig. 4c) and *Rest* expression (Fig. 4d) is inversely correlated in exocrine cells and in β-cells. A high expression of *Scrt1* in β-cells was observed, while *Rest* was expressed only in exocrine cells. Next REST was overexpressed in the mouse insulinoma cell line MIN6 and gene expression was analysed by qPCR for *Rest*, *Ins2*, *Pdx1*, *Snap25* and *Scrt1* three days post infection (Fig. 4e). While *Ins2* and *Pdx1* (not direct targets of REST) were unaffected by *Rest* overexpression, *Snap25* (a known target of REST^40^) and *Scrt1* expression were significantly reduced. Opposite results were obtained in primary exocrine cells infected with Ad-mCherry control or Ad-DN-REST virus (encoding a dominant negative REST protein^43^). At 5 days post-infection, *Scrt1* expression was significantly higher when *Rest* was silenced using the dominant negative mutant (Fig. 4f). These results are consistent with a REST-mediated repression of the *Scrt1* gene. Next, we examined *Scrt1* and *Rest* expression levels in a model of β-like cells undergoing reprogramming from primary exocrine cells 3 and 6 days post-infection^6,15^. A continuous accumulation of *Scrt1* mRNA and a parallel reduction in *Rest* were observed (Fig. 4g,h). Lastly, *Rest* overexpression in FACS-sorted reprogrammed cells resulted in a marked decrease in *Scrt1* expression (Fig. 4i). Taken together, our results demonstrate that *Scrt1* is controlled by REST. Thus, REST represses *Scrt1* in exocrine cells and *Scrt1* increases during reprogramming in parallel with a concomitant decline in *Rest* expression.

## Discussion

Postnatal islet maturation is a critical process to achieve proper β-cell function. Immediately after birth, β-cells are not fully functional and have to undergo a major gene reprogramming to acquire the ability to secrete adequate amounts of insulin in response to glucose^44^. Our group and others^7,23,30,45^ have shown a large-scale rewiring of transcriptional programs occurring during the neonatal period. However, little is known on cis-regulation of gene expression at the chromatin level before and after weaning. Chromatin accessibility of human islets on a genome-wide scale has been previously produced using FAIRE-seq^31,46^. More recently, several studies took advantage of ATAC-seq together with GWAS to identify causal variants of T2D in cis-regulatory elements in human^47–50^. Another study identified cell-type specific accessible sites and transcription factor binding sites in α, β and acinar cells^12^. However, the transcriptional regulation of postnatal islet maturation at the chromatin level has not been reported so far. In this project, we employed ATAC-seq^13,51^ to produce a global map of accessible sites (ACS) in the islets of 10-day-old pups and in adult rats. This permitted to detect more than 100,000 ACS, among which about 20% were differentially accessible before and after the functional maturation of β-cells. Interestingly, the ACS with the most significant p-value and a large fold-change was located on the 3′UTR of the *Mrs2* gene. This gene is encoding a magnesium transporter at the surface of the mitochondria^52^. Genetic variants in this magnesium-related ion channel were previously associated with type 2 diabetes and pancreatic cancer^53,54^.

Using two different computational approaches and the Jaspar PWM database, we could find known and unforeseen transcriptional regulators potentially involved in the maturation process. We identified many DNA-binding proteins affecting chromatin accessibility, including MAF, FOX, FOS/JUN, NRF, E2F, CTCF, RFX, SREB, NKX6, REL, MEIS, TEAD and SCRT1. Several of these transcription factors have been already implicated in pancreas development and postnatal islet maturation^55^. For instance, E2F1 is an established cell survival and proliferation activator in β-cells^28^ and we have recently shown that this transcription factor controls the expression of the long non-coding RNA H19 and is profoundly down-regulated during the postnatal period. Moreover, we obtained evidence suggesting that E2F1 and H19 may contribute to the decrease in β-cell mass in the maternal low protein diet offspring model^45^.

Next, we integrated these accessibility maps with mRNA micro-array data from^30,45^ in the same model and we found that ~ 60% of the differentially accessible sites were nearby differentially expressed genes, suggesting that these cis-regulatory elements are involved in the transcriptional regulation of gene expression. Several pathways previously related to the maturation process such as insulin secretion or circadian rhythms are under the control of these accessible cis-regulatory elements. For instance, several components of the core clock are not rhythmic in 10 days old pups but are consistently oscillating in β-cells after weaning^56^. It has been recently demonstrated that circadian rhythms trigger islet maturation via clock-controlled metabolic cycles^27^. These cis-regulatory elements may be controlled by transcriptional enhancers or repressors. Indeed, we confirmed that 3 out of the 5 tested ACS that are located nearby genes important for proper β-cell function had a significant enhancer activity. These ACS were located nearby *Syt4, Neurod1*, and *Mafb*. We showed that an enhancer site and the promoter of *Mafb* depicted a decreased accessibility along maturation. *Mafb* is required at the early stage of β-cells maturation and decreases in later stages^32^. We observed that an enhancer near *Neurod1* was significantly more accessible and *Neurod1* expression was higher in mature β-cells. Another group showed that overexpression of *Mafa* results in increased *Neurod1* expression^57^, and that *Mafa* is crucial to induce glucose-responsive insulin secretion in neonatal rat β-cells. We showed that *Syt4* is a potential target of NFAT, FOX and RFX TFs family (Supplementary Fig. S5). *Syt4* expression was correlated with its enhancer accessibility and was higher in fully mature β-cell. *Syt4* is known to modulate Ca^2+^ sensitivity of insulin granules and consequently enhance GSIS^58^.

One of the identified transcriptional regulators, SCRT1, is of particular interest. Scrt1 was previously shown to be implicated in brain development^33,34^ but its role in β-cells remained to be determined. While this manuscript was under revision, Scrt1 was suggested to act as a regulator of insulin expression and secretion in response to glucose in combination with a cAMP raising agent (IBMX)^59^. Here, downregulation of Scrt1 in adult rat islets did not impact insulin release in response to only high concentration of glucose but induced β-cell proliferation, suggesting that reduced expression of *Scrt1* in P10 islets may favour the acquisition of an appropriate β-cell mass during the neonatal period. Indeed, transcripts related to proliferation were significantly impacted by the downregulation of *Scrt1* in FACS-sorted adult β-cells. Interestingly, a significant increase of *Scrt1* in adult islet cells was also reported in a recent study analyzing age-dependent gene expression and chromatin changes in human^60^. RNA-seq analyses suggest that *Scrt1* affects *Syt4*, *Notch1* and *Nfatc1* and *Nfatc2* gene expression and consequently may control the calcineurin/NFAT pathway, an important regulator of β-cell growth and function^61,62^. Functional enrichment analysis showed that *Scrt1* also influences the expression of genes related to oxygen sensing and autophagy. Accordingly, previous studies have demonstrated a role for the hypoxia-inducible factor HIF1a in normal β-cell function^63^, and altered β-cell autophagy in human T2DM^64^.

Intriguingly, Ackermann and others^5,12^ pointed out that many poised genes in *α* cells contain a signature of functional β-cells and, consequently, could be of use for *α*-to-β cell reprogramming. This suggests plasticity and specialization of the different cell types along maturation and rewiring of transcriptional programs at the chromatin level. In fact, reprogramming of acinar cells into insulin-producing cells has been accomplished using adenoviral gene delivery of PDX1, NGN3 and MafA^6^. A subsequent study characterized these changes using RNA-seq and ChIP-qPCR and revealed that loss of *Rest* combined with *Pdx1* expression leads to activation of endocrine genes and correlates with epigenetic modifications of local chromatin^15^. In this study, we provide evidence indicating that *Scrt1* is a direct target of REST in different mouse and human cell models. Moreover, overexpression of *Rest* inhibits Scrt1 expression in β-cells and in β-like cells reprogrammed from exocrine cells. Islet plasticity is of interest for the design of future treatments of diabetes through the production of mature surrogate insulin-producing cells^65^. Thus, understanding the role of *Scrt1* in the reprogramming process will be important to improve the generation of mature surrogate insulin-producing cells. REST downregulation has been suggested to be involved in postnatal β-cell maturation in response to thyroid hormones^57^ and it is tempting to speculate that *Scrt1* derepression might also contribute to this process.

In this study, ATAC-seq and the mRNA microarray analyses have been performed in whole islets and not in FACS-sorted cells or in single cells. Several recent studies have demonstrated a large heterogeneity of gene expression at a single cell level using smFISH or single cell RNA-seq^23,66–68^. Taking advantage of the heterogeneity of gene expression together with single cell ATAC-seq could shed more light on the transcriptional control of islet maturation and the different cell types involved.

**Figure 5:**
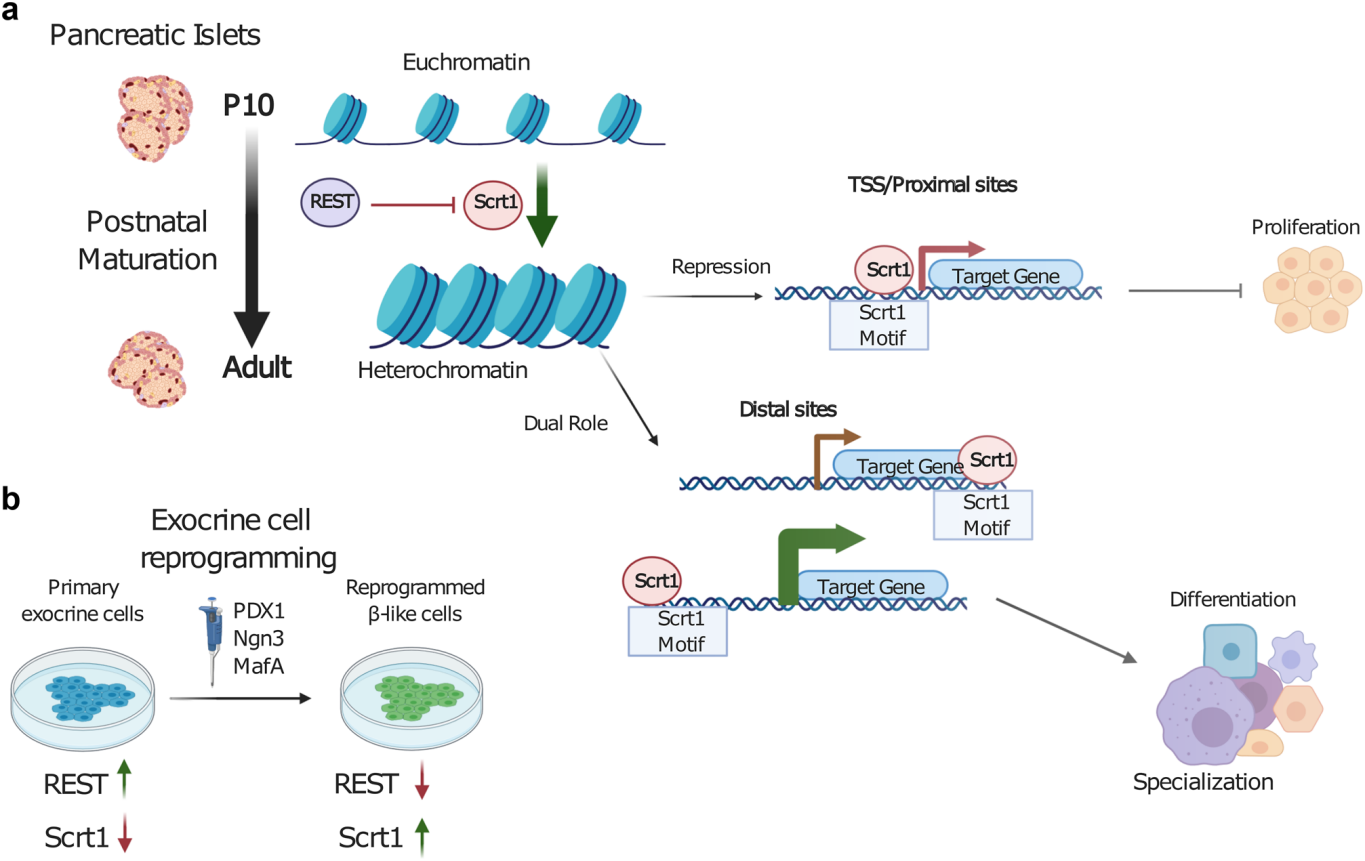
Summary figure: Chromatin accessibility along maturation is affected by Scrt1. In the present study we investigated the chromatin accessibility along the post-natal pancreatic islet maturation process. (**a**) SCRT1 motif is enriched in ACS that are closing upon maturation. We found that *Scrt1* is a direct target of REST and that the Scrt1 motif is present in TSS/proximal sites as well as distal sites. The inhibition of Scrt1 lead to increased proliferation and has additional effects on other processes such as specialization. (**b**) In an in vitro reprogramming system, *Scrt1* expression raises after PNM infection and is anti-correlated with REST levels.

Overall, we produced a high-resolution map of chromatin accessible sites in islets of 10-day-old pups and adult rats (Fig. 5a). These genome wide accessibility maps are an important resource to study cis-regulation of gene expression along islet cell maturation. Using these maps, we discovered a new important transcriptional repressor implicated in the maturation process, namely *Scrt1*, which controls β-cell proliferation and function. *Scrt1* is controlled by REST and its expression is increased along acinar to β-cell reprogramming (Fig. 5b). Manipulations of the level or the activity of this transcriptional regulator may favour the development of new approaches aiming at generating surrogate insulin-secreting cells. A global understanding of the molecular mechanisms and of the transcription factors involved in functional maturation will be seminal for the design of β-cell-based replacement strategies for the treatment of diabetes.

## Methods and materials

### Animals

Male (200-250 g, around P90) and pregnant Sprague–Dawley rats were obtained from Janvier laboratories (Le Genest St- Isle, France). After birth, male and female pups were nursed until sacrifice at P5, P10, P20 or P23. Animals were housed under standard temperature (20-24ºC) and humidity (40–70%) conditions on a 12 h light/dark cycle with free access to chow diet and water. All procedures were performed in accordance with the Guidelines for the care and use of laboratory animals from the National Institutes of Health and of ARRIVE and were approved by the Swiss Research Councils and Veterinary Office. All animal experiments involving reprogramming of exocrine cells isolated from C57BL/6JOlaHsd or MIP-GFP mice, were approved by the Weizmann Institute Institutional Animal Care and Use Committee.

### Islet isolation, dispersion and sorting

Islets were isolated by collagenase digestion of the pancreas^69^. In more details, P5 and P10 pancreases of 3–5 pups were collected, pooled and digested by hand shaking in 3 ml of collagenase P solution (Sigma) at 1 mg/ml for 3–5 min, while 3 or 10 ml of collagenase P solution were injected in the pancreatic duct of P20–23 pup and adult rats, respectively, followed by 8 and 15 min digestion in a warm bath. After digestion, islets were separated from the exocrine tissue using an histopaque density gradient followed by hand-picking and were incubated for 2 h in RPMI 1640 GlutaMAX medium (Invitrogen) containing 11 mM glucose and 2 mM l-glutamine and supplemented with 10% fetal calf serum (Gibco), 10 mM Hepes pH 7.4, 1 mM sodium pyruvate, 100 μg/mL streptomycin and 100 IU/mL penicillin. Dissociated islet cells were obtained by incubating the islets in Ca^2+^/Mg^2+^ free phosphate buffered saline, 3 mM EGTA and 0.002% trypsin for 5 min at 37 °C. For some experiments, islet cells were separated by Fluorescence-Activated Cell Sorting (FACS) based on β-cell autofluorescence, as previously described^70,71^. Sorted islet cells were seeded on plastic dishes coated with extracellular matrix secreted by 804 G rat bladder cancer cells (804 G ECM)^72^. Enrichment of *α*- and β-cells was evaluated by double immunofluorescence staining using polyclonal guinea pig anti-insulin (dilution 1:40, PA1-26938 Invitrogen) and polyclonal mouse anti-glucagon (dilution 1:1000, Abcam Ab10988) antibodies, followed by goat anti-guinea pig Alexa-Fluor-488 and goat anti-mouse Alexa-Fluor-555 (diluted 1:400, Thermofisher A11073 and A21422, respectively) secondary antibodies. On average, β-cell fractions contained 99.1 ± 0.9% insulin-positive cells and 0.6 ± 0.6% glucagon-positive cells and α-cell-enriched fractions contained 10.6 ± 8.2% insulin-positive cells and 88.8 ± 8.2% glucagon-positive cells.

### ATAC-seq sample preparation

ATAC-seq libraries were prepared as previously described^51^ using dissociated islet cells from 3 adult male rats and from a mix of few P10 pups of 3 different litters. Briefly, 100′000 islet cells were resuspended in 50 μl of cold lysis buffer (10 mM Tris–HCl pH7.4, 10 mM NaCl, 3 mM MgCl2 and 0.1% IGEPAL CA-630) and centrifuged at 500 g for 10 min at 4 °C. The pellet was resuspended in the transposase reaction mix (Nextera kit, Illumina). The transposition reaction was performed at 37 °C for 30 min and was followed by purification of the samples using the Qiagen MinElute PCR purification kit (Qiagen). Transposed DNA fragments were amplified for 11 cycles using the NEBnext high-fidelity PCR master mix and the Ad1_noMX and Ad2.1–2.6 barcoded primers from^51^. Amplified libraries were purified with AMPure XP beads (Beckman Coulter) to remove contaminating primer dimers. Library quality was assessed using the Fragment Analyzer and quantitated using Qubit. All libraries were sequenced on Illumina HiSeq 2500 using 100 bp paired-end reads.

### ATAC-seq data quality control and analysis

Fastq files quality was assessed using FastQC (version 0.11.2)^73^. Raw reads were aligned to the Rattus norvegicus reference genome assembly 5 (Rn5) using BWA (version 0.7.13)^74^ with default settings. Quality control of the aligned reads was checked using Samstat (version 1.5)^75^ and processed with Samtools (version 1.3)^76^. Reads mapping to mitochondrial DNA were discarded from the analysis together with low quality reads (MAPQ < 30). Peak calling was performed in order to find accessible sites (ACSs) using Macs2 (version 2.1.1)^17^ on adult and P10 samples concatenated separately. ACSs were then reunited in a single bed file and quantified using the pyDNase library (version 0.2.5)^77^ (Table 1). We used FIMO^20^ from the MEME suite (version 4.11.4) together with Jaspar 2016 position-weight matrices^21^, to predict transcription factor binding sites. Finally, we used the R statistical software (version 3.4.2) and several bioconductor and CRAN packages to perform gene sets enrichment analysis (RDavidWebService, ClusterProfiler)^78^, ACSs localization analysis (ChIPseeker)^19^, motif enrichment analysis (FGSEA, Supplementary Table 2)^22^ and penalized linear model analysis (GLMnet, Supplementary Table 3)^29^ for motif selection. All sequencing tracks were viewed using the Integrated Genomic Viewer (IGV 2.4.8)^79^. ATAC-seq raw data were deposited in GEO under the accession number GSE122747.

### mRNA microarray

P10 and adult mRNA expression from^30,45^ were reanalyzed with EdgeR and RDavidWebservice^18,80^ (supplementary Table 6). These microarray data are available in the GEO database under the accession number GSE106919.

### Cell line

The INS 832/13 rat β-cell line was provided by Dr. C. Newgard (Duke University)^81^ and was cultured in RPMI 1640 GlutaMAX medium (Invitrogen) containing 11 mM glucose and 2 mM L-glutamine and supplemented with 10% fetal calf serum (Gibco), 10 mM Hepes pH 7.4, 1 mM sodium pyruvate and 0.05 mM of β-mercaptoethanol. INS 832/13 cells were cultured at 37 °C in a humidified atmosphere (5% CO_2_, 95% air) and tested negative for mycoplasma contamination. MIN6 cells were a kind gift from Prof. J. Miyazaki (Osaka University). Min6 cells were culture in DMEM medium containing 11 mM d‐glucose supplemented with 15% fetal calf serum, 200 IU/ml penicillin, 100 μg/ml streptomycin, 2 μM l‐glutamine, and 72 μM β-mercaptoethanol. For experiments involving infections with Ad-hREST adenovirus, 300,000 cells per well were plated on 24-well plates and were harvested 72 h post infections.

### Cell transfection

Dispersed rat islet cells or FACS-sorted β-cells were transfected with a pool of 4 siRNAs directed against rat Scrt1 or a negative control (On-Target plus 081299-02 and 001810-10 respectively, Dharmacon) using Lipofectamine RNAiMAX (Thermofisher). INS 832/13 cells were co-transfected with pGL3 promoter and psicheck plasmids (Promega) using lipofectamine 2000 (Thermofisher). pGL3 promoter vector was empty (control) or contained an enhancer region for *MafB, NeuroD1, Pax6* or *Syt4* (RNA synthesis, subcloning and plasmid sequencing were performed by GenScript, Netherlands) (Supplementary Table 4). RNA extraction and functional assays were performed 48 h after transfection.

### Luciferase assay

Luciferase activities were measured in INS 832/13 cells using the Dual-Luciferase Reporter Assay System (Promega). Firefly luciferase activity was normalized to Renilla luciferase to minimize experimental variabilities. Experiments were performed in triplicates.

### RNA extraction, quantification and sequencing

RNA was extracted using miRNeasy micro kit (Qiagen) followed by DNase treatment (Promega). Gene expression levels were determined by qPCR using miScript II RT and SYBR Green PCR kits (Qiagen) and results were normalized to the housekeeping gene *Hprt1*. Data were analyzed using the 2^−ΔΔ*C*(*T*)^ method. Primer sequences are provided in Table 2. For mRNA-sequencing, the RNA was converted into a sequencing library using the Illumina TruSeq RNA-sequencing kit and standard Illumina protocols. Single-end, 151 nt long reads were obtained using a HiSeq 4000 instrument. Reads were aligned to the transcriptome (Rnor6) with Kallisto^82^ and presented as transcripts per million (TPM) and EST pseudo counts (Suppementary table 5). Subsequently, differential expression was calculated using Sleuth^83^. Finally, biological function and pathway analyses were performed using Cluster profiler^78^. RNA-seq raw data were deposited in the GEO database under the accession number GSE130651.

### Insulin secretion and content

Transfected rat islet cells were pre-incubated for 30 min at 37 °C in Krebs–Ringer bicarbonate buffer (KRBH) containing 2 mM glucose, 25 mM HEPES, pH 7.4 and 0.1% BSA (Sigma-Aldrich), followed by 45 min incubation at 2 or 20 mM glucose. At the end of the incubation period, media were collected for insulin determination and rat islet cells were lysed with acid–ethanol (0.2 mM HCl in 75% ethanol) to extract total insulin content or with protein lysis buffer to measure total protein content (Bradford, BioRad). The amount of insulin released in the medium and remaining in the cells was measured by insulin Elisa kit (Mercodia). All experiments were performed in triplicates.

### Cell death assay

TUNEL staining on rat β-cells was performed 48 h after transfection using the TMR red In Situ Cell Death Detection Kit (Roche) combined to polyclonal guinea pig anti-insulin (dilution 1:40, PA1-26938 Invitrogen) followed by incubation with goat anti-guinea-pig AlexaFluor 488 antibody (dilution 1:400, A11073 Thermofisher). Cell nuclei were stained with Hoechst 33342 (1 μg/ml, Invitrogen). Coverslips were mounted on microscope glass slides with Fluor-Save mounting medium (VWR International SA) and were visualized with a Zeiss Axiovision fluorescence microscope. A minimum of 10^3^ cells were counted per condition. Incubation for 24 h with a mix pro-inflammatory cytokines (1 ng/mL IL-1β, 10 ng/mL TNF-*α* and 30 ng/mL IFN-γ) was used as positive control. Experiments were performed in single replicates.

### Proliferation assay

The proliferative capacity of the beta-cells was assessed by BrdU incorporation. Transfected islet cells were cultured on poly-l-lysine coated glass coverslips for a total of 72 h and BrdU (Ab142567, Abcam) at 10 µM final concentration was added to the culture media for the last 48 h. Cells were fixed with ice cold methanol and permeabilized with 0.5% (wt/vol) saponin (Sigma-Aldrich) and DNA was denatured with 2 N HCl. The coverslips were first incubated with monoclonal mouse anti-BrdU (dilution 1:400, BD55627, BD Biosciences) and polyclonal guinea pig anti-insulin (dilution 1:40, PA1-26938, Invitrogen) antibodies followed by incubation with goat anti-mouse Alexa-Fluor-555 (dilution 1:400, A21422 Thermofisher) and goat anti-guinea-pig Alexa-Fluor-488 (dilution 1:400, A11073 Thermofisher) antibodies. At the end of the incubation, nuclei were stained with Hoechst 33342 (Invitrogen). Coverslips were mounted on microscope glass slides with Fluor-Save mounting medium (VWR International SA) and were visualized with a Zeiss Axiovision fluorescence microscope. Images of at least 10^3^ cells per condition were collected. Incubation with Prolactin (PRL 500 ng/ml during 48 h) was used as positive control. Experiments were performed in single replicates.

### Exocrine to β cell reprogramming

The experimental procedure of Acinar to β-cell reprogramming and for islet and β cell isolation was carried as described^15,84^. Briefly, primary exocrine cells were isolated from Mip-GFP mice^85^. Exocrine cells were cultured and infected by Ad-M3-mCherry (expressing PDX1, NGN3 MafA^86^). Day 3 and 6 cells were FACS-sorted to isolate mCherry and GFP fractions.

### Adenovirus production, amplification and infections

The adenoviral plasmid coding for Ad-M3-mCherry was obtained from Addgene (Addgene #61041, Qiao Zhou). The mCherry adenoviral plasmid was a generous gift from Prof. Qiao Zhou (Harvard Stem Cell Institute). The DN-REST construct was a generous gift from Prof. Gail Mandel (Oregon Health and Science University) and adenoviral plasmid construction was performed using AdenoQuick2.0 system (O.D.260 Inc.). The human REST adenovirus^87^ was a generous gift from Dr. David Martin, Dr. Florent Allagnat and Prof. Jacques-Antoine Haefliger, Lausanne University Hospital. Adenoviral generation, amplification and infections were carried as previously described^84^. Exocrine cell infections were carried out immediately following cell isolation, in 6-well plates using exocrine-cell clusters containing 850 µg protein per well, in 2 mL of culture media containing the following virus titers: Ad-M3-mCherry-10*10^6^ IFU/ml; Ad-hREST-10*10^6^ IFU/ml; Ad-DN-REST-30*10^6^ IFU/ml. 24 h following infection, cells were washed using culture media, and cells were plated on collagen-coated plates. MIN6 cells were infected with Ad-hREST or Ad-mCherry control at MOI 500. 24 h following infection, cells were washed using culture media and harvested for downstream analysis by qPCR 72 h post infection. Primer sequences for qPCR assays are provided in Table 3.

### Data analysis of RNA-seq from reprogrammed β-like cells

To analyze *Scrt1* and *Rest* expression in exocrine, β-, and β-like reprogrammed cells RNA-seq data from GEO (GSE128545) was used. Analysis was carried using UTAP (UTAP: User-friendly Transcriptome Analysis Pipeline^88^, https://utap.readthedocs.io/en/latest/). Normalized counts of Scrt1 and REST were used to analyze their expression level in exocrine (Cherry−/GFP−), M3 infected (Cherry + /GFP−), reprogrammed β-like enriched (Cherry + /GFP +) and β cells (GFP + from islets of MIP-GFP mice).

### ChIP-seq data analysis

To show transcription factor occupancy near the Scrt1 locus bigWig files were uploaded to IGV^79^ mm10 genome assembly: mouse NKX6-1 (GEO: GSE40975)^38^ (islets), mouse INSM1, FOXA2 and NeuroD1 (GEO: GSE54046)^36^(Jia et al., 2015) (islets), mouse H3K4Me3 (GEO: GSE63020)^39^ (islets), mouse REST (GEO: GSE27148)^42^ (mES cells), and human REST (GEO: GSE32465)^89^ (hES cells & PANC-1 cells) were downloaded from the Cistrome database^90^ (http://cistrome.org/db/). Raw data (fastq sequence files) for mouse PDX1 binding in islets^37^ was obtained (Array Express, E-MTAB-1143) and analyzed by mapping against the mm10 with bowtie.

### Statistical analysis

Data are expressed as mean ± SD. Statistical significance was determined using parametric unpaired two-tailed Student’s t-test or, for multiple comparisons, with one-way analysis of variance (ANOVA) of the means, followed by post-hoc Dunnett or Tukey test (Graph Pad Prism6). P-values less than 0.05 (p < 0.05) were considered statistically significant**.**

### Schematic drawing

Schemes were created with the web application of BioRender.com.

## Supplementary Information

Supplementary Table 1.

Supplementary Table 2.

Supplementary Table 3.

Supplementary Table 4.

Supplementary Table 5.

Supplementary Table 6.

Supplementary Table 7.

Supplementary Table 8.

Supplementary Table 9.

Supplementary Figures.

## Data Availability

ATAC-seq raw data were deposited in GEO under accession number GSE122747. Microarray data from^30,45^ are available in the GEO database under accession number GSE106919. RNA-seq raw data were deposited in the GEO database under accession number GSE130651.
