# Supplementary material for: Scrt1, a transcriptional regulator of β-cell proliferation identified by differential chromatin accessibility during islet maturation
Source: Sci Rep. 2021 Apr 22;11:8800. doi: 10.1038/s41598-021-88003-2 (PMC8062533; doi:10.1038/s41598-021-88003-2)
Supplement: Supplementary file 10 — Supplementary Figures. [file 41598_2021_88003_MOESM10_ESM.pdf]

Supplementary information

Scrt1, a transcriptional regulator of β-cell proliferation identified by differential chromatin accessibility during islet maturation

Jonathan Sobel[1,2], Claudiane Guay[1], Ofer Elhanani[2], Adriana Rodriguez-Trejo[1], Lisa Stoll[1,3], Véronique Menoud[1], Cécile Jaccovetti[1], Michael D. Walker[2] and Romano Regazzi[1,4]

[1]Department of Fundamental Neurosciences, University of Lausanne, Rue du Bugnon 9, 1005 Lausanne, Switzerland

[2]Department of Biomolecular Sciences, Weizmann Institute of Science, Rehovot 7610001, Israel

[3]current address: Department of Medicine, Weill Cornell Medicine, 413 East 69th Street, New York, NY, 10021, USA

[4] Department of Biomedical Sciences, University of Lausanne, Rue du Bugnon 7, 1005 Lausanne, Switzerland

Supplementary Tables

(1) ATAC-seq P10 versus Adult islet cells: All annotated ACS detected in pancreatic islet cells. Significantly differentially expressed genes in P10 vs Adult islet cells nearby significantly changing ACS, and KEGG pathway analysis using David of P10, Adult and Stable ACS annotated with the name of the closest gene.

(2) FGSEA for motif accessibility. Table includes motif name, p-value, FDR, enrichment and normalized enrichment score, most extreme ACS rank, and number of target ACS significantly changing.

(3) Penalised linear model GLMnet for inference of transcription factor binding site motif activity. ACS sequences were scanned in order to construct the matrix of motifs per ACS. Every ACS with their respective $\log_2$ accessibility fold change (Adult/P10) were used to infer the motif activity, called β (see Methods). If β is negative, the motif is more accessible in P10 sites, while if β is positive the motif explains a higher accessibility of the ACS in adults. Our linear model uses a penalty λ of 0.007 and an α of 0.1. The table includes motif name, inferred accessibility coefficient β, motif consensus sequence, motif information content, and number of ACS targets

(4) Validated ACS luciferase assay: Accessible sites information and sequences used in the luciferase assay to assess enhancer activity.

(5) SiScrt1 versus Ctrl RNA-seq of β-cells. The file contains the read counts per genes, and the statistical analysis of the differential gene expression performed with Sleuth.

(6) Microarray of P10 versus adult rat pancreatic islet cells. The file contains the probe intensities and the statistical analysis of the differential gene expression performed with edgeR.

(7) Genes differentially expressed in siScrt1 versus control adult rat β-cells (RNA-seq) and in adult versus P10 rat islets (microarray)

(8) Gene ontology of biological process enriched in differentially expressed genes in siScrt1 versus control adult rat β-cells and in adult versus P10 rat islets

(9) RNA-seq of Ctrl β-cells and of FAC-sorted day 3 and 6 exocrine cells expressing M3-mCherry or NGN3-GFP.

Supplementary Figure Legends

Fig. S1. ATAC-seq quality control. (**a**) Fragment size distribution for each sample. (**b**) Correlation heatmap of samples. (**c**) Principal component analysis of each ATAC-seq sample. (**d**) Genome-wide localisation and classification of ACS: localisation of 100,000 random peaks compared to true accessible sites. True ACS are enriched in intronic and exonic regions. (**e**) Localisation of stable ACS, ACS more accessible in adult islet cells (Up) and ACS more accessible in P10 islet cells (Down). ACS more accessible in adults are enriched in introns, while stable and ACS more accessible in P10 are enriched in promoter regions.

Fig. S2. A distal enhancer located 12Kbp away from *Mafb* is less accessible upon maturation and correlates with Mafb expression. (**a**) IGV screenshot of the *Mafb* locus with four accessible sites detected. Two of them are significantly less accessible upon maturation (highlighted with red rectangles), notably the site at the TSS of *Mafb* and an enhancer located 12kb away. Several motifs such as MEIS2/3, GLEIS2, Klf4, have been detected in the distal enhancer and are potentially responsible for the decreased accessibility of this site. mRNA level of (**b**) *Mafb* and (**c**) *Scrt1* expressed as normalized counts from the RNA-seq data of Qiu et al. [23] in mouse along pancreatic *β*-cell maturation. (**d**) *Scrt1* expression in rat islets measured by qPCR and normalized to the housekeeping gene *Hprt1* along pancreatic *β*-cell maturation. One-way ANOVA Dunnett's post hoc test $^{**}p< 0.01$.

Fig. S3. Representative images of apoptosis and proliferation assays. (a) Representative images of Tunel staining (Fig. 2**i**) of dispersed adult rat islet cells transfected with a control siRNA (siCtl) or siRNAs directed against Scrt1 (siScrt1). Tunel assay was performed 48h after transfection in basal (NT) condition or in response to a mix (cyt mix) of pro-inflammatory cytokines (IL-1β, TNF-α, IFN-γ). White arrows indicate apoptotic (Tunel+, red) β-cells (insulin+, green). **(b)** Representative images of BrdU staining (related to Fig. 2**j**) of dispersed adult rat islet cells transfected with a control siRNA (siCtl) or siRNAs directed against Scrt1 (siScrt1). BrdU staining was performed 72h after transfection in basal (NT) condition or in response to prolactin (PRL). White arrows indicate proliferative (BrdU+, red) β-cells (insulin+, green).

Fig. S4. Downregulation of *Scrt1* expression in FAC-sorted *β*-cells. The level of the indicated genes was measured by qPCR in sorted adult rat *α*- and/or *β*-cells and normalized to *Hprt1* level. (**a**) *Scrt1* expression in sorted *α*- and *β*-cells. (**b**) *Scrt1* expression in FAC-sorted *β*-cells 48h after transfection with a control siRNA

(siCtl) or with siRNAs directed against Scrt1 (siScrt1). (**c**) Insulin and (**d**) glucagon expression in FAC-sorted *α*- and *β*- cell fractions. * $p < 0.05$ by Student's t-test

Fig. S5. Enhancer motif content and gene expression along maturation. (**a**) Nfatc2 Enhancer accessibility in P10 and Adult with underlying the transcription factor binding motifs present. (**b**) Nfatc2 mRNA expression measured in FAC-sorted pancreatic *β*-cells of adult rats treated with siSCRT1 and control. (**c**) *Nfatc2* expression in mouse islets along maturation from [23]. (**d**) Syt4 Enhancer accessibility with TFBS motif content. (**e**) Syt4 expression in siScrt1 and siCtrl treated *β*-cells, and in P10 or adult islets. (**f**) Syt4 expression along maturation in mouse islets. (**g**) Notch1 Promoter accessibility with TFBS motif content. (H) Notch1 expression in siScrt1 and siCtrl treated *β*-cells, and in P10 or adult islets. (**i**) Notch1 expression along maturation in mouse islets. (**j**) Neurod1 Enhancer accessibility with TFBS motif content. (**k**) Neurod1 expression in siScrt1 and siCtrl treated *β*-cells, and in P10 or adult islets. (**l**) Neurod1 expression along maturation in mouse islets. mRNA expression in siScrt1 versus siRNA control (ctl) adult rat *β*-cells were measured by RNA-seq and in P10 versus adult rat islets using micro-array, * $p < 0.05$, ** $p < 0.01$, *** $p < 0.001$. ACS significantly more accessible in adult are highlighted with green rectangles while less accessible sites are depicted with red rectangles.

Fig. S6. Srct1 promoter bound by REST in hES cells and PANC-1 cells. REST Chip-seq data in human Embryonic Stem (hES) cells and in the human pancreatic cell line PANC-1 [89] was downloaded from cistromedb and visualised using the IGV genome browser.

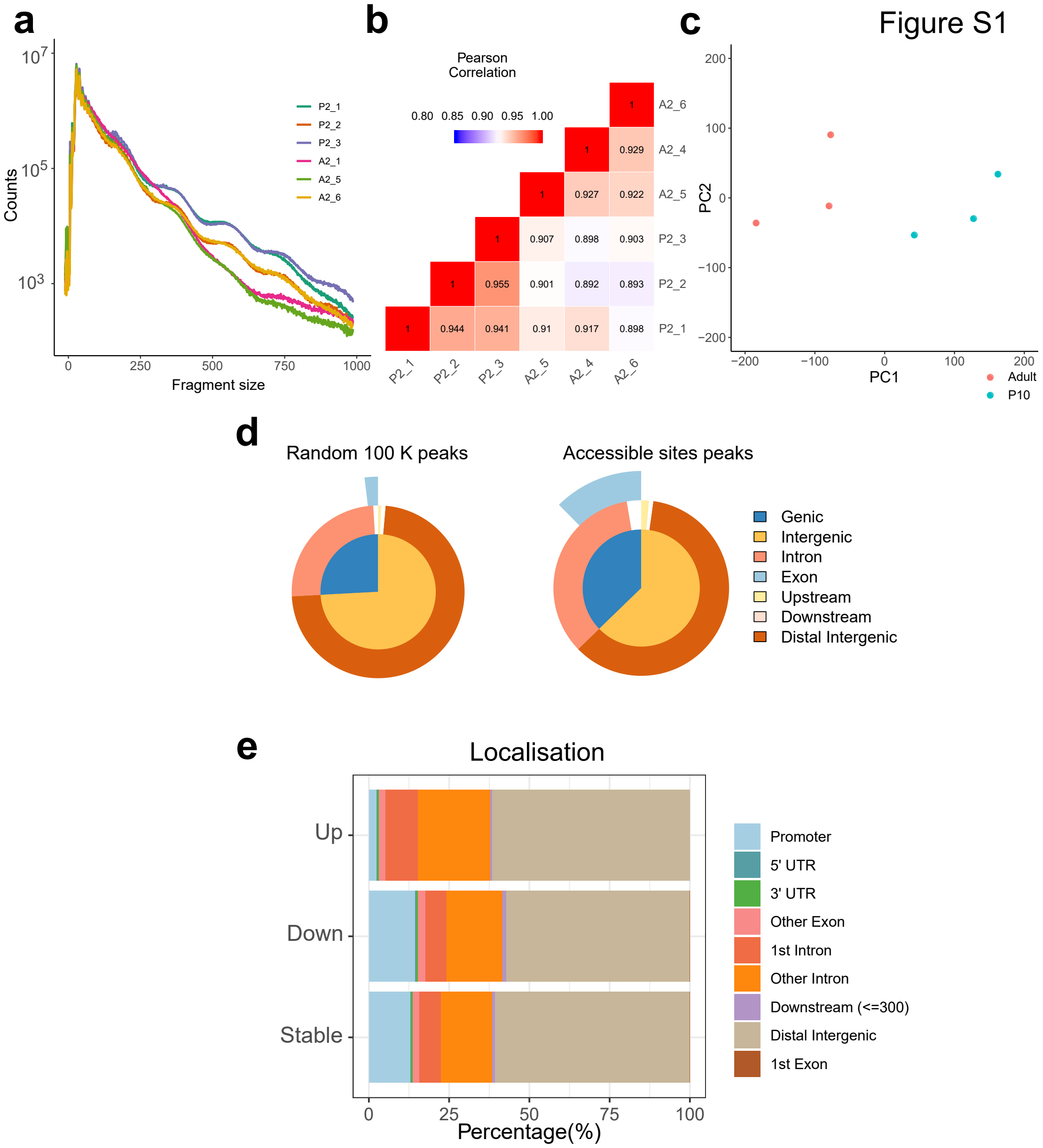
Figure S1
a
Counts
Fragment size
P2_1
P2_2
P2_3
A2_1
A2_5
A2_6
b
Pearson Correlation
0.80 0.85 0.90 0.95 1.00
c
PC1
PC2
Adult
P10
d
Random 100 K peaks
Accessible sites peaks
Genic
Intergenic
Intron
Exon
Upstream
Downstream
Distal Intergenic
e
Localisation
Up
Down
Stable
Percentage(%)
Promoter
5' UTR
3' UTR
Other Exon
1st Intron
Other Intron
Downstream (<=300)
Distal Intergenic
1st Exon

Figure S2

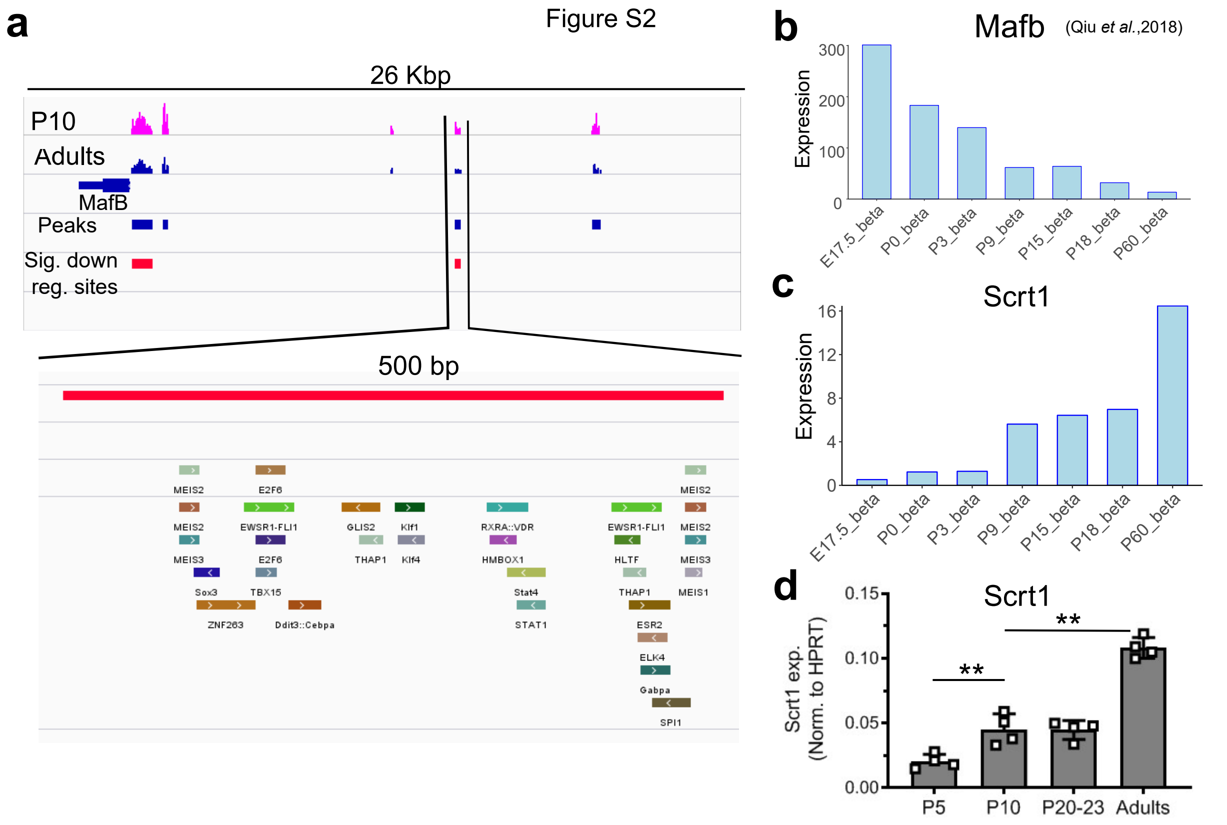
a
26 Kbp
P10
Adults
MafB
Peaks
Sig. down reg. sites
500 bp
MEIS2
E2F6
MEIS2
MEIS2
EWSR1-FLI1
GLIS2
Klf1
RXRA::VDR
EWSR1-FLI1
MEIS2
MEIS3
E2F6
THAP1
Klf4
HMBOX1
HLTF
MEIS3
Sox3
TBX15
Stat4
THAP1
MEIS1
ZNF263
Ddit3::Cebpa
STAT1
ESR2
ELK4
Gabpa
SPI1
b
Mafb
(Qiu et al.,2018)
Expression
0
100
200
300
E17.5_beta
P0_beta
P3_beta
P9_beta
P15_beta
P18_beta
P60_beta
c
Scrt1
Expression
0
4
8
12
16
E17.5_beta
P0_beta
P3_beta
P9_beta
P15_beta
P18_beta
P60_beta
d
Scrt1
Scrt1 exp.
(Norm. to HPRT)
0.00
0.05
0.10
0.15
**
**
P5
P10
P20-23
Adults

Figure S3A

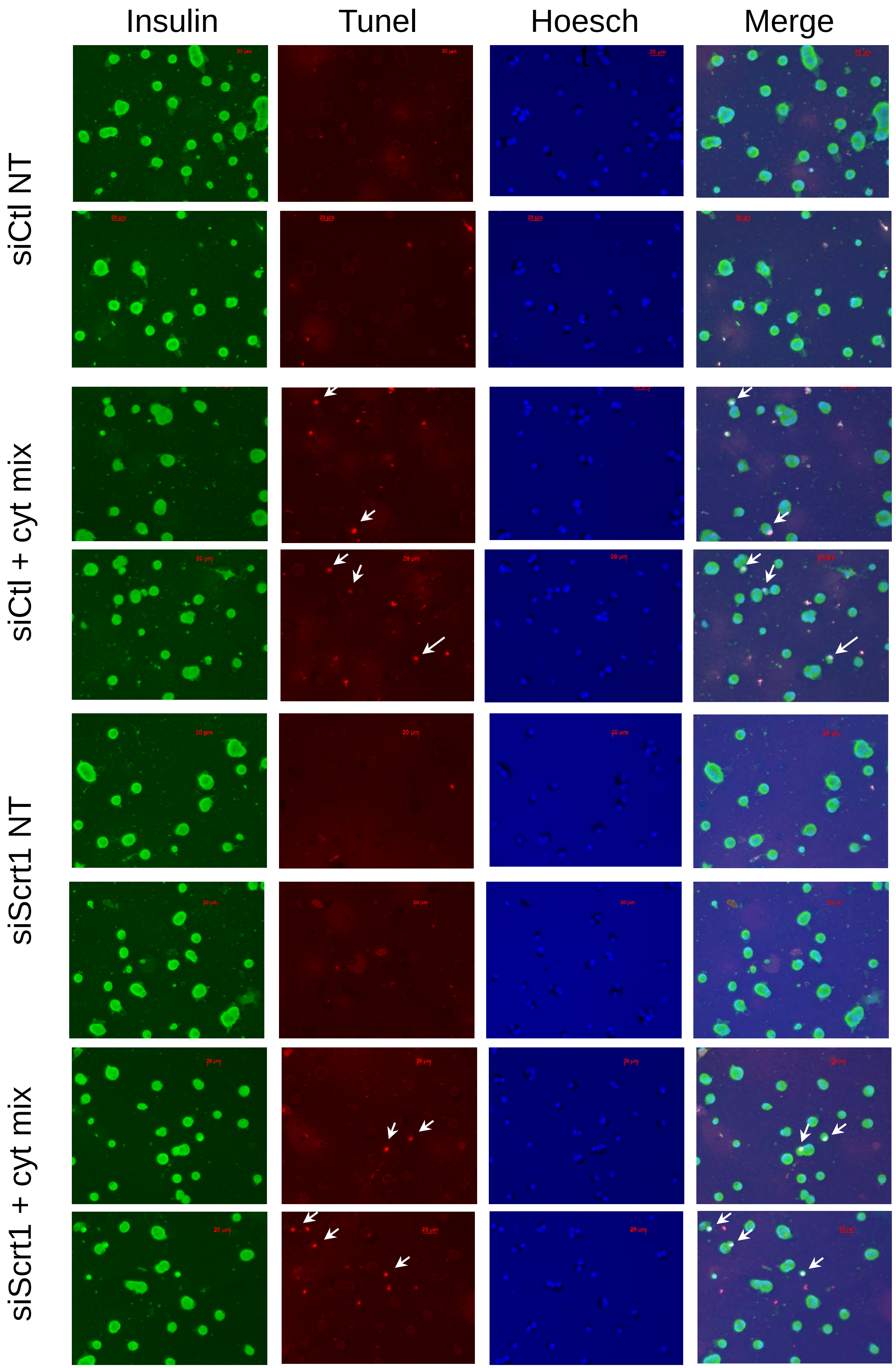

Insulin
Tunel
Hoesch
Merge
siCtl NT
siCtl + cyt mix
siScrt1 NT
siScrt1 + cyt mix

Figure S3B

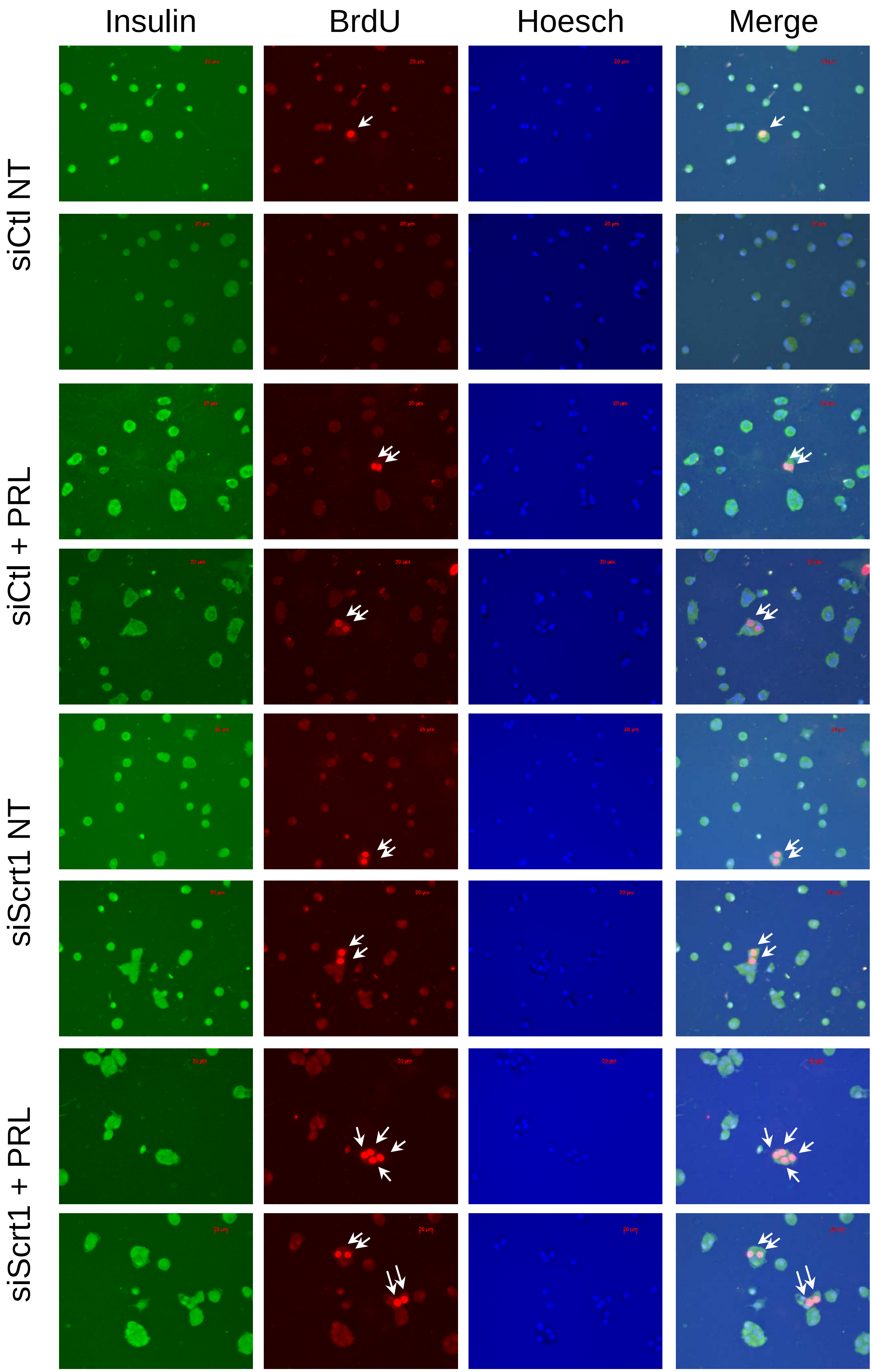
Insulin
BrdU
Hoesch
Merge
siCtl NT
siCtl + PRL
siScrt1 NT
siScrt1 + PRL

Figure S4

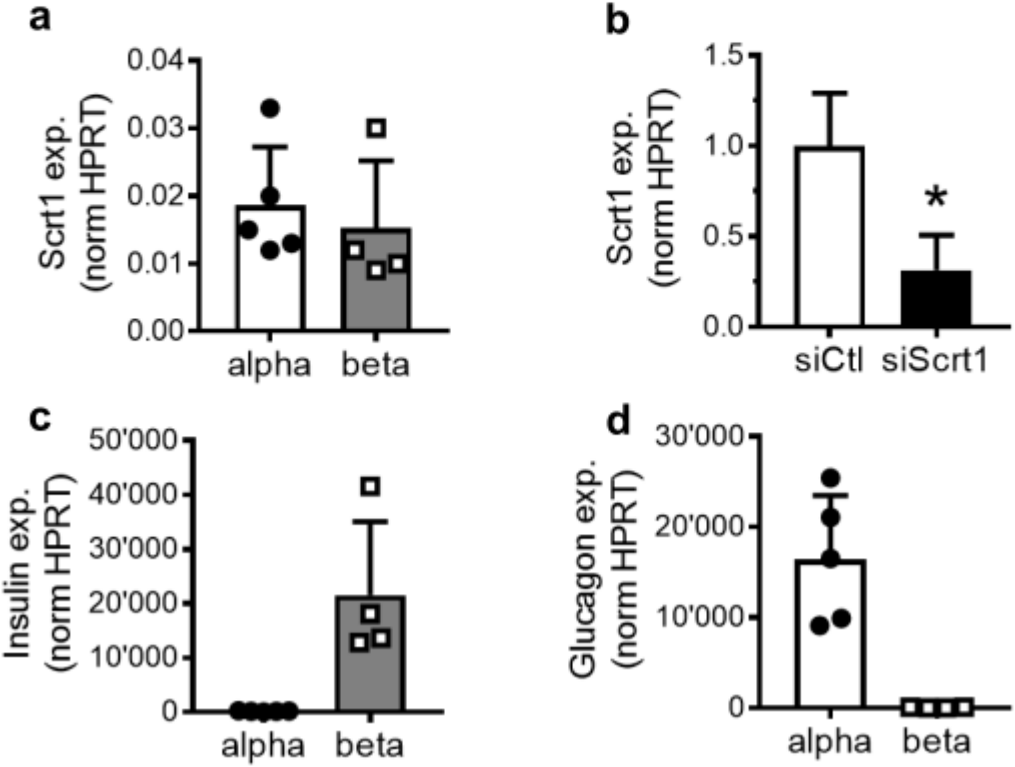
a
0.04
0.03
0.02
0.01
0.00
Scrt1 exp.
(norm HPRT)
alpha
beta
b
1.5
1.0
0.5
0.0
Scrt1 exp.
(norm HPRT)
*
siCtl
siScrt1
c
50'000
40'000
30'000
20'000
10'000
0
Insulin exp.
(norm HPRT)
alpha
beta
d
30'000
20'000
10'000
0
Glucagon exp.
(norm HPRT)
alpha
beta

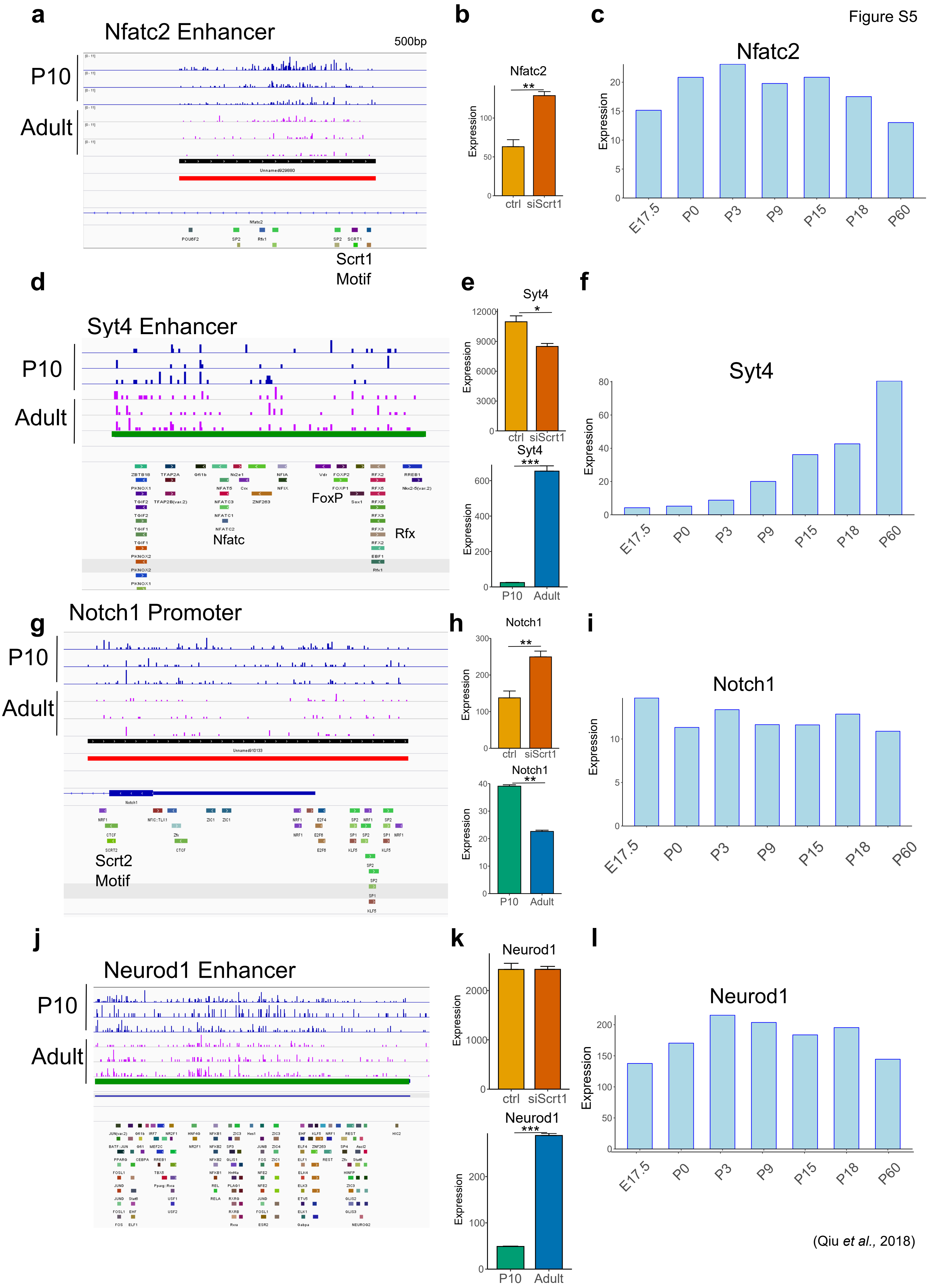

(Qiu *et al.*, 2018)

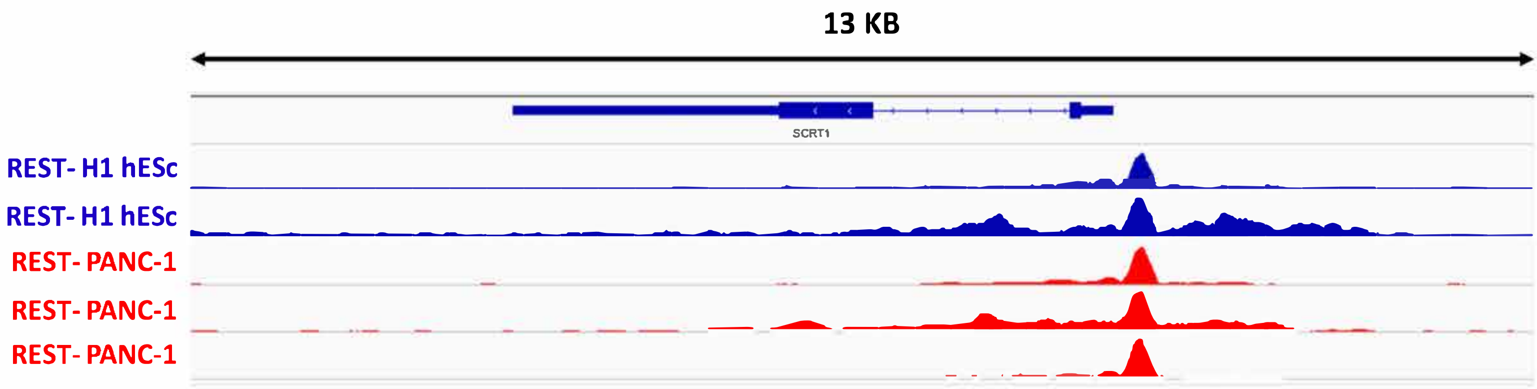
13 KB
SCRT1
REST- H1 hESc
REST- H1 hESc
REST- PANC-1
REST- PANC-1
REST- PANC-1

Figure S6
